# Supplementary material for: The molecular evolution of spermatogenesis across mammals
Source: Nature. 2022 Dec 21;613(7943):308–16. doi: 10.1038/s41586-022-05547-7 (PMC9834047; doi:10.1038/s41586-022-05547-7)
Supplement: Supplementary file 1 — Supplementary Figs. 1–5, which show technical aspects of QC and ISH quantifications, and the legends for Supplementary Tables 1–11 (in a separate Excel file). [file 41586_2022_5547_MOESM1_ESM.pdf]

nature portfolio

https://doi.org/10.1038/s41586-022-05547-7

Supplementary information

The molecular evolution of spermatogenesis across mammals

In the format provided by the
authors and unedited

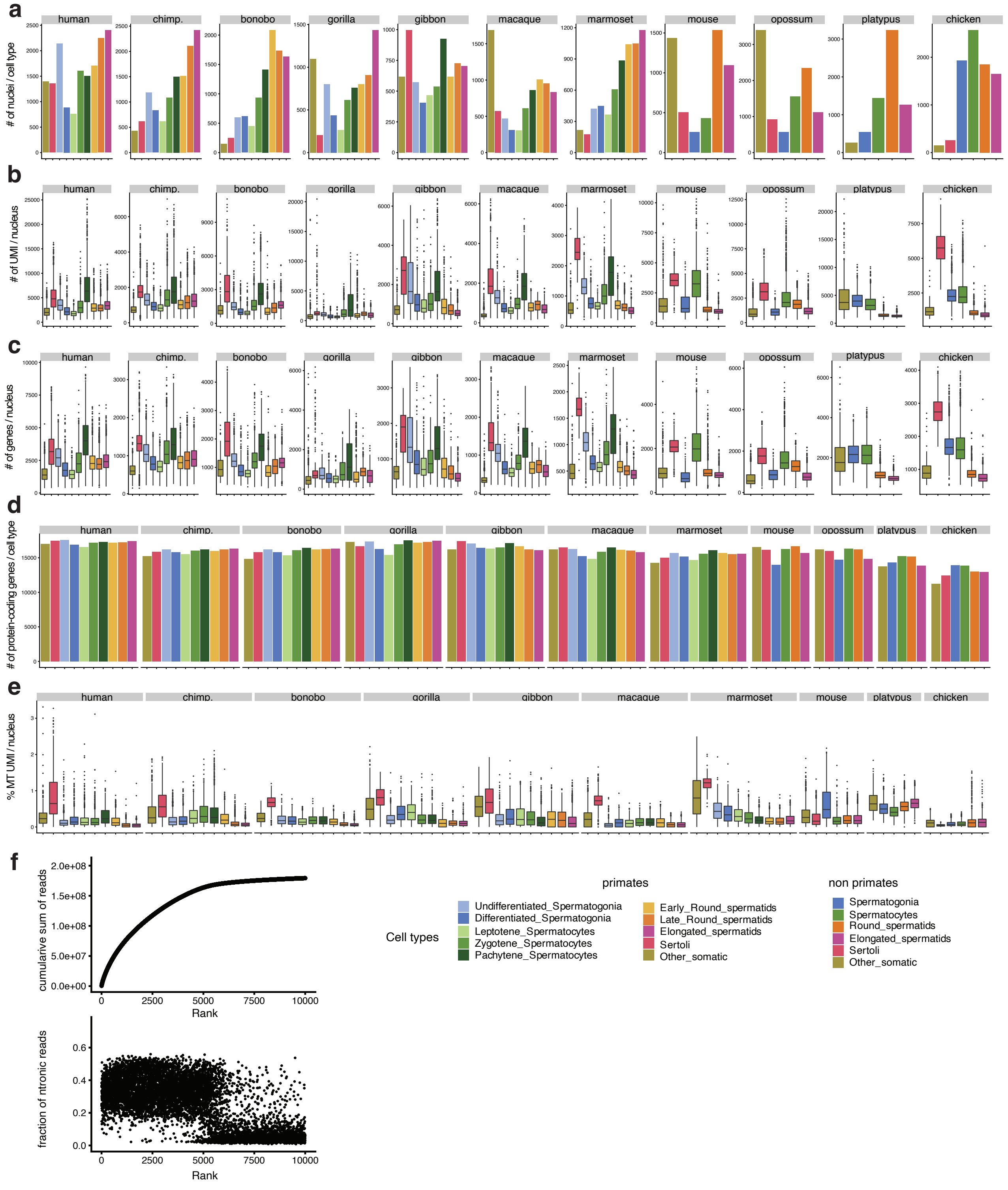

Supplementary Fig. 1 | Data overview and quality controls across cell types and species. a, Numbers of nuclei per cell type. **b**, Numbers of UMI per nucleus. **c**, Numbers of genes per nucleus. **d**, Numbers of protein-coding genes per cell type. **e**, Percentages of mitochondrial UMIs per nucleus. **f**, Cumulative sum of reads per droplet (top) and fraction of intronic reads per droplet (bottom) for one human individual (human.2.2); droplets are ranked by the number of reads (decreasing order). b, c, e, Box plots depict the median (center value); upper and lower quartile (box limits) with whiskers at 1.5 times the interquartile range.

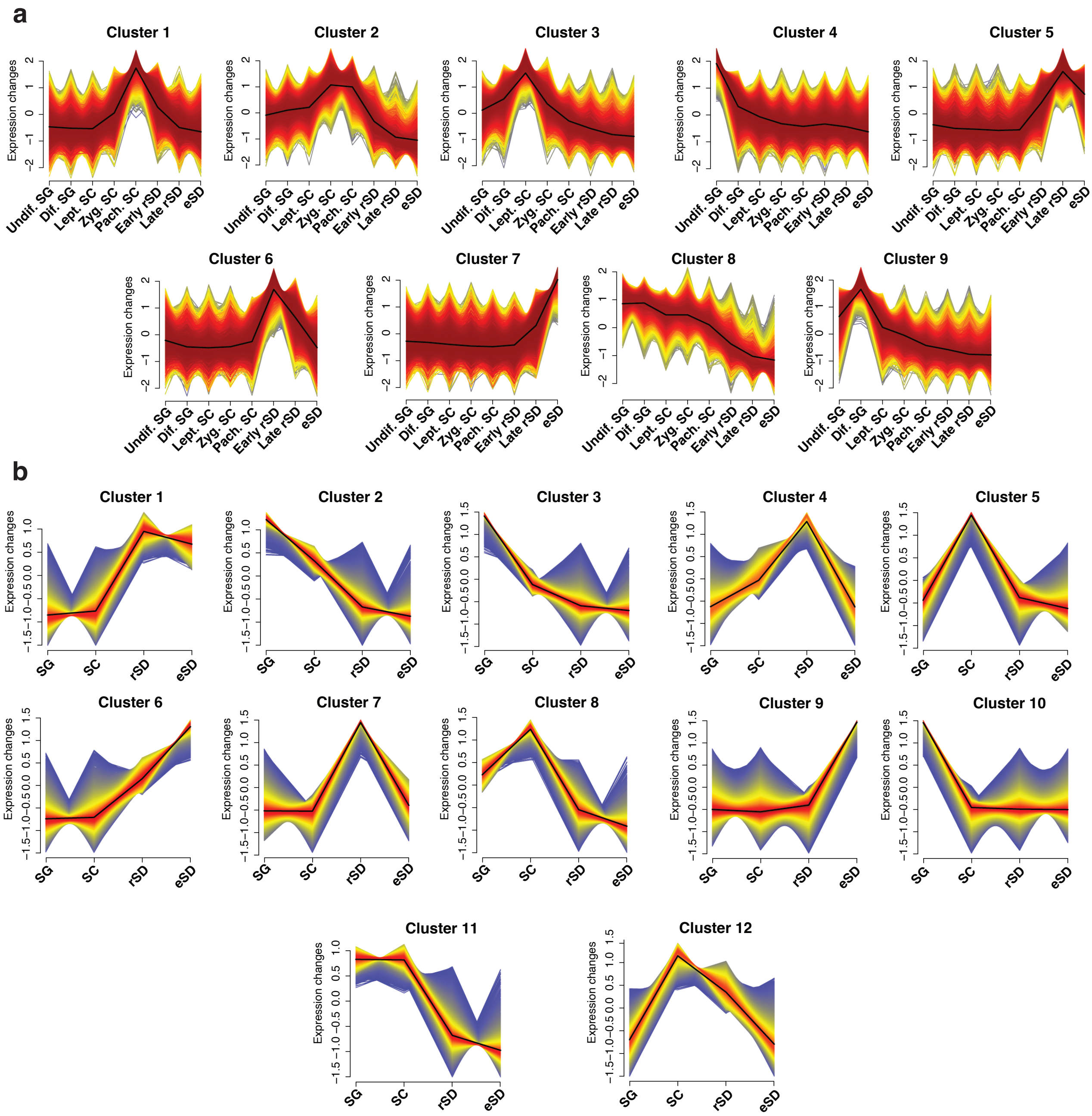

Supplementary Fig. 2 | Gene expression trajectory clusters. **a**, Gene expression trajectory clusters for primates (human, chimp., bonobo, gorilla, gibbon, macaque, marmoset). **b**, Gene expression trajectory clusters for amniotes (human, mouse, opossum, platypus, chicken).

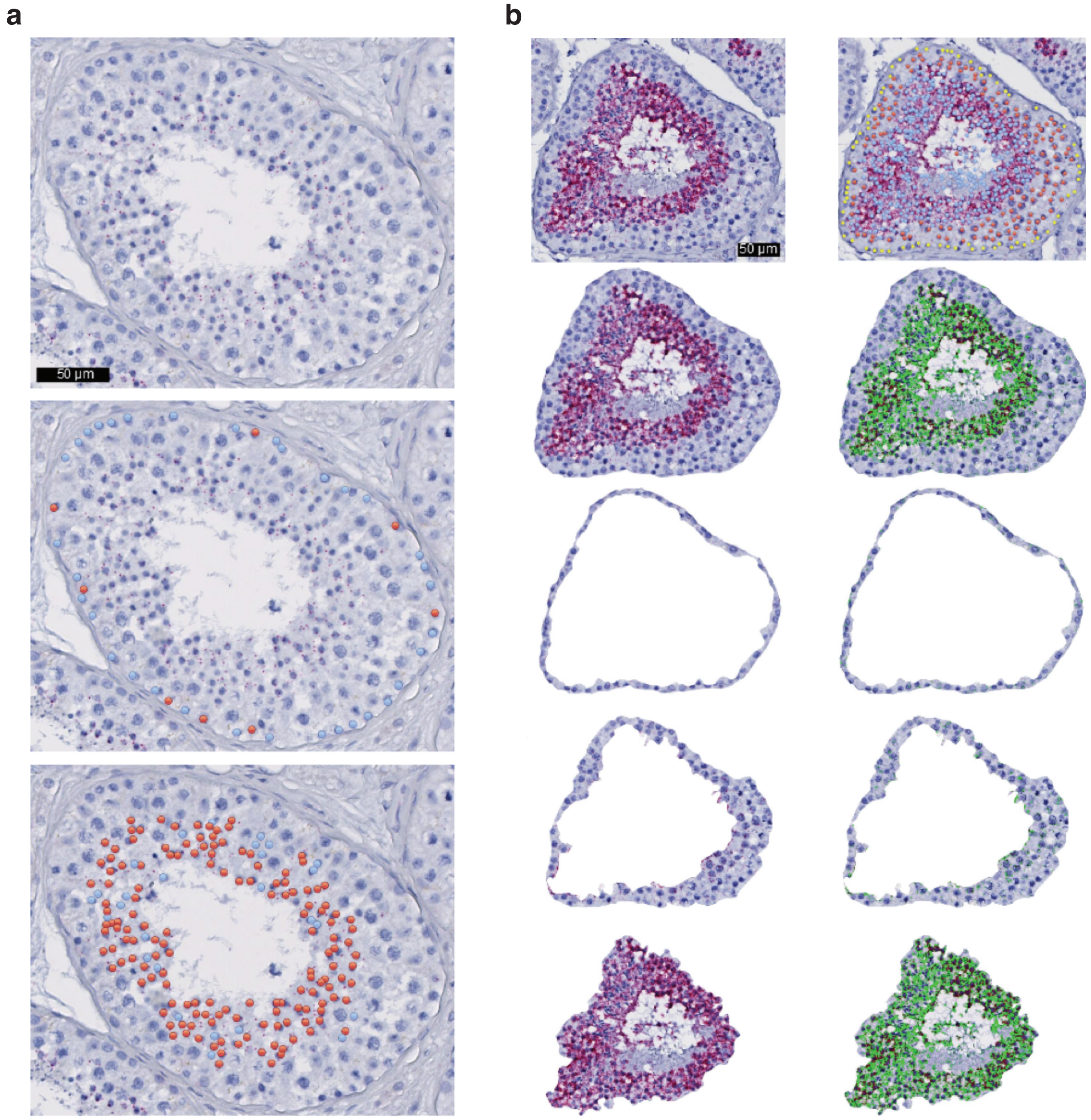

Supplementary Fig. 3 | Quantification of gene expression from smISH experiments. a, Example of *MYO3B* staining and expression quantification in an orangutan seminiferous tubule. Dots show *MYO3B* positive (red) and negative (blue) spermatogonia (middle) and round spermatids (bottom). **b**, Example of *RUBCNL* staining and expression quantification in a chimpanzee seminiferous tubule. Single-molecule RNA staining is shown in red and computationally detected transcripts are colored in green. The first row shows *RUBCNL* staining (left) and the three main spermatogenic cell types (spermatogonia, in yellow; spermatocytes, in red; and spermatids, in blue). The second, third, fourth and fifth rows show *RUBCNL* staining (left) and detected transcripts (right) at the whole tubule, spermatogonia, spermatocytes and spermatids levels, respectively.

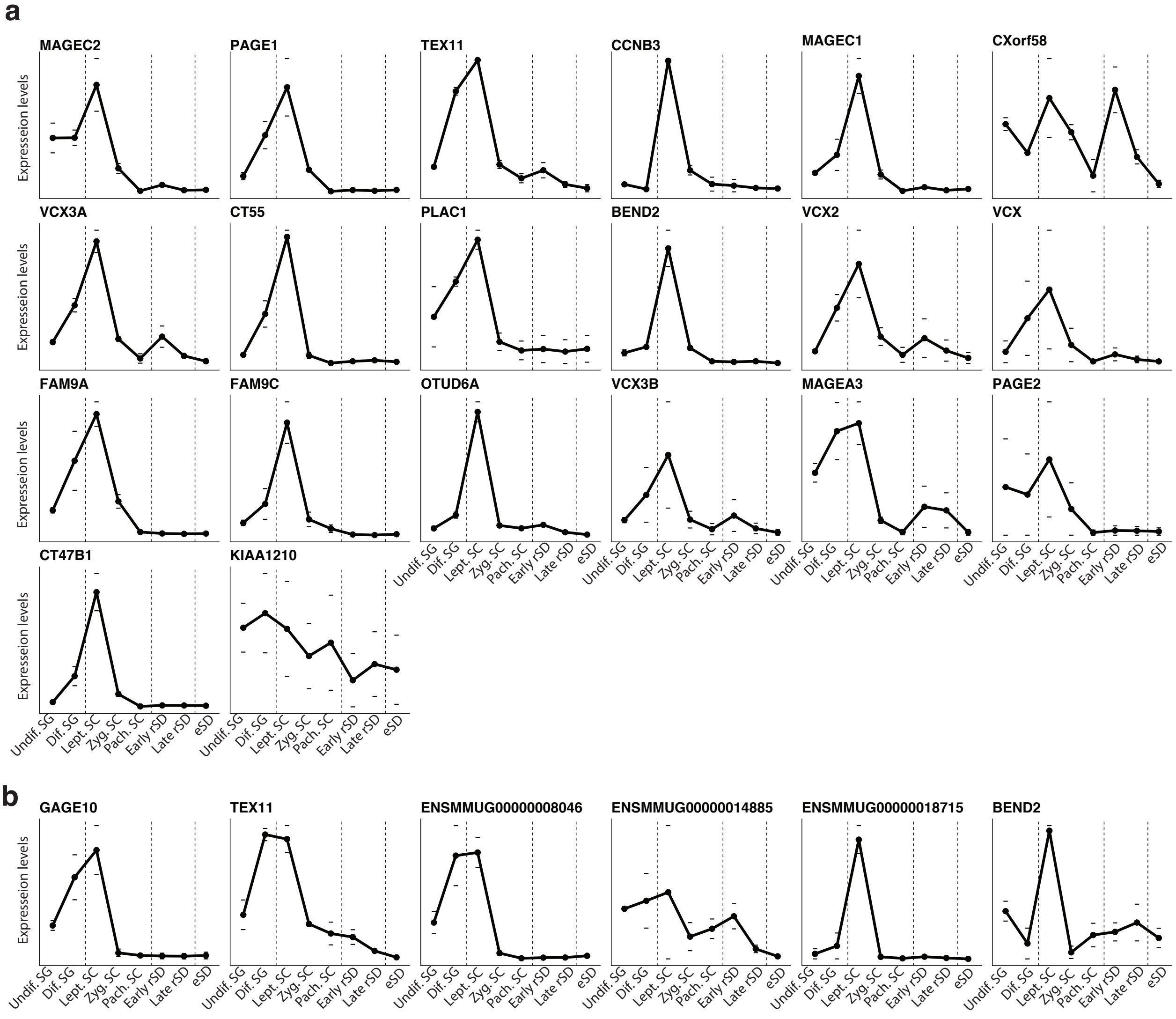

Supplementary Fig. 4 | Trajectories. Mean expression of testis-specific X-linked genes with predominant expression in leptotene spermatocytes in human (**a**) and macaque (**b**) along spermatogenesis. The marks indicate the minimum and maximum mean expression values of the two biological replicates, dots indicate the median of these values.

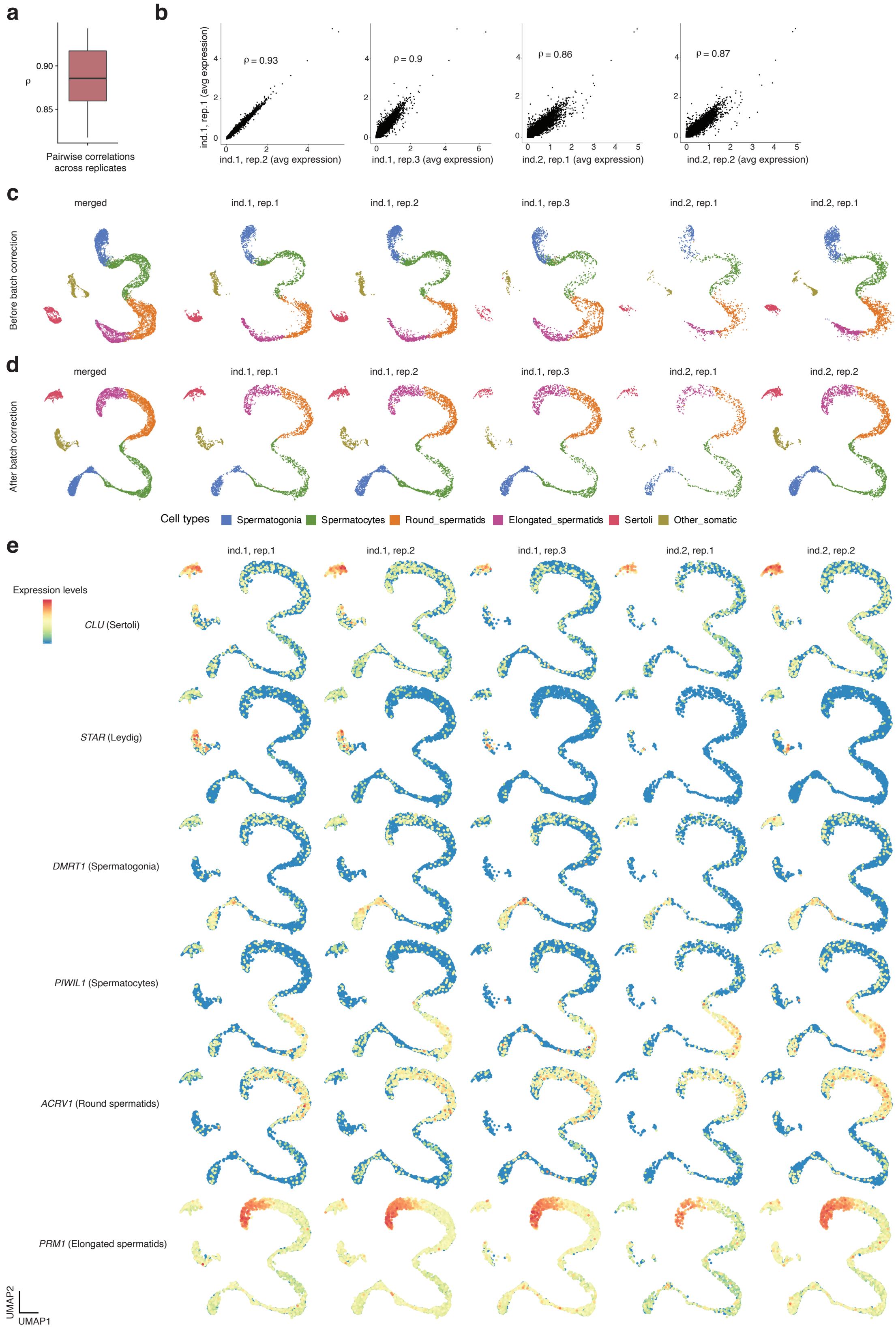

Supplementary Fig. 5 | Batch comparisons. a, Distribution of Spearman correlation coefficients (ρ) for pairwise comparisons between replicates for all species (n = 27). Box plot depicts the median (center value); upper and lower quartile (box limits) with whiskers at 1.5 times the interquartile range. **b**, Comparisons of gene expression levels (mean level for each gene across all cells of the sample) between human ind.1/rep.1 and the other replicates. **c**, UMAP of merged human samples before batch correction (left) and individual UMAPs of each independent human sample using the merged coordinates (right). **d**, UMAP of merged human samples after batch correction (left) and UMAPs of each independent human samples using the merged UMAP coordinates (right). **e**, Marker gene expression across human replicates using the merged UMAP coordinates after batch correction.

Legends for Supplementary Tables 1-11

Supplementary Table 1: Description of the 34 libraries generated in this study

Supplementary Table 2: Testis-specific genomic annotation for each species

Supplementary Table 3: Single-nucleus features

Supplementary Table 4: Cell type marker genes for each species. External gene names are flanked by underscores in order to avoid any excel formatting issues.

Supplementary Table 5: Conserved and changed trajectories across primates. Columns H-T show the trajectory cluster for each species and ancestor. Columns U-Z show conserved trajectories. Columns AA-AF show the probability that identifies if trajectories between two groups of species are similar (< 0.05 implies a change). Columns AG-AL indicate in which lineage the change occurred.

Supplementary Table 6: In situ hybridization. Top: Experimental conditions for each probe in each of the three different species. Bottom: Probe information

Supplementary Table 7: Conserved and changed trajectories across amniotes. Columns F-N show the trajectory cluster for each species and ancestor. Columns O-R show conserved trajectories. Columns S-V show the probability that identifies if trajectories between two groups of species are similar (< 0.05 implies a change). Columns W-Z indicate in which lineage the change occurred.

Supplementary Table 8: Mouse infertility genes, emergence and expression peak

Supplementary Table 9: Significant interacting pairs involving Sertoli cells across human, macaque, mouse, opossum and chicken

Supplementary Table 10: Per cell type testis specific genes for human, macaque, mouse, opossum, platypus and chicken

Supplementary Table 11: Significantly enriched genes in X-bearing and Y-bearing spermatids
