# Supplementary material for: The molecular evolution of spermatogenesis across mammals
Source: Nature. 2022 Dec 21;613(7943):308–16. doi: 10.1038/s41586-022-05547-7 (PMC9834047; doi:10.1038/s41586-022-05547-7)
Supplement: Supplementary file 2 — Reporting Summary [file 41586_2022_5547_MOESM2_ESM.pdf]

nature portfolio

Corresponding author(s): Florent Murat, Noe Mbengue, Henrik Kaessmann

Last updated by author(s): Nov 2, 2022

Reporting Summary

Nature Portfolio wishes to improve the reproducibility of the work that we publish. This form provides structure for consistency and transparency in reporting. For further information on Nature Portfolio policies, see our Editorial Policies and the Editorial Policy Checklist.

Statistics

For all statistical analyses, confirm that the following items are present in the figure legend, table legend, main text, or Methods section.

n/a	Confirmed	
☐	☒	The exact sample size (n) for each experimental group/condition, given as a discrete number and unit of measurement
☐	☒	A statement on whether measurements were taken from distinct samples or whether the same sample was measured repeatedly
☐	☒	The statistical test(s) used AND whether they are one- or two-sided<br>*Only common tests should be described solely by name; describe more complex techniques in the Methods section.*
☒	☐	A description of all covariates tested
☐	☒	A description of any assumptions or corrections, such as tests of normality and adjustment for multiple comparisons
☐	☒	A full description of the statistical parameters including central tendency (e.g. means) or other basic estimates (e.g. regression coefficient) AND variation (e.g. standard deviation) or associated estimates of uncertainty (e.g. confidence intervals)
☐	☒	For null hypothesis testing, the test statistic (e.g. F, t, r) with confidence intervals, effect sizes, degrees of freedom and P value noted<br>*Give P values as exact values whenever suitable.*
☒	☐	For Bayesian analysis, information on the choice of priors and Markov chain Monte Carlo settings
☒	☐	For hierarchical and complex designs, identification of the appropriate level for tests and full reporting of outcomes
☐	☒	Estimates of effect sizes (e.g. Cohen's d, Pearson's r), indicating how they were calculated

Our web collection on statistics for biologists contains articles on many of the points above.

Software and code

Policy information about availability of computer code

Data collection	No software was used.
Data analysis	Open source software including CellRanger (2.1.1) and (3.0.2), CellPhoneDB v2, cutadapt (1.8.3), Tophat2 (2.1.1), StringTie (1.3.3), cuffcompare (v2.2.1), PAML (4.9), NDP.view2Plus (2.8.24), the R (3.6.2) packages Seurat (3.1.4), biomaRt (2.40.5), LIGER (0.5.0), slingshot (1.2.0), mfuzz (2.44.0), ape (5.3), dplyr (0.8.5), countcolors (0.9.1), colordistance (1.1.1), mixtools (1.2.0), ggplot2 (3.2.1), tidyverse (1.3.0), cowplot (1.0.0), limma (3.40.6), UpSetR (v1.4.0), and pheatmap (1.0.12) were used in this study for data analyses.

For manuscripts utilizing custom algorithms or software that are central to the research but not yet described in published literature, software must be made available to editors and reviewers. We strongly encourage code deposition in a community repository (e.g. GitHub). See the Nature Portfolio guidelines for submitting code & software for further information.

Data

Policy information about availability of data

All manuscripts must include a data availability statement. This statement should provide the following information, where applicable:

- Accession codes, unique identifiers, or web links for publicly available datasets
- A description of any restrictions on data availability
- For clinical datasets or third party data, please ensure that the statement adheres to our policy

Raw and processed bulk and single-nucleus RNA-seq data have been deposited in ArrayExpress with the accession codes E-MTAB-11063 (human snRNA-seq), E-MTAB-11064 (chimpanzee snRNA-seq), E-MTAB-11067 (bonobo snRNA-seq), E-MTAB-11065 (gorilla snRNA-seq), E-MTAB-11066 (gibbon snRNA-seq), E-MTAB-11068 (macaque snRNA-seq), E-MTAB-11069 (marmoset snRNA-seq), E-MTAB-11071 (mouse snRNA-seq), E-MTAB-11072 (opossum snRNA-seq), E-MTAB-11070 (platypus snRNA-seq), E-MTAB-11073 (chicken snRNA-seq) and E-MTAB-11074 (chimpanzee, gorilla, gibbon and marmoset bulk RNA-seq) (https://

www.ebi.ac.uk/arrayexpress/).
IMPC database is available at: ftp://ftp.ebi.ac.uk/pub/databases/impc/
Receptor-ligand interactions: https://www.cellphonedb.org/downloads
Gene phylogenetic ages: http://gentree.ioz.ac.cn/
Translational efficiency data: E-MTAB-7247
Time and tissue specificity data: https://apps.kaessmannlab.org/evodevoapp/
pLI scores: http://genetics.bwh.harvard.edu/genescores/selection.html

Field-specific reporting

Please select the one below that is the best fit for your research. If you are not sure, read the appropriate sections before making your selection.

☒ Life sciences ☐ Behavioural & social sciences ☐ Ecological, evolutionary & environmental sciences

For a reference copy of the document with all sections, see nature.com/documents/nr-reporting-summary-flat.pdf

Life sciences study design

All studies must disclose on these points even when the disclosure is negative.

Sample size	No statistical methods were used to determine sample size. Sample size was based on the number of individuals available (see Supplementary Table 1).
Data exclusions	Low quality nuclei were excluded as described in Methods.
Replication	We generated 2 biological replicates for human (5 technical replicates), bonobo, gorilla, macaque, marmoset, mouse, platypus, and chicken; 3 biological replicates for chimpanzee and opossum; and 2 technical replicates for gibbon.
Randomization	Not relevant, because no treatment groups.
Blinding	Blinding was not relevant to our study. Both data collection and analyses required an understanding of the nature of the sample being collected/analyzed.

Reporting for specific materials, systems and methods

We require information from authors about some types of materials, experimental systems and methods used in many studies. Here, indicate whether each material, system or method listed is relevant to your study. If you are not sure if a list item applies to your research, read the appropriate section before selecting a response.

Materials & experimental systems

n/a	Involved in the study	
☒	☐	Antibodies
☒	☐	Eukaryotic cell lines
☒	☐	Palaeontology and archaeology
☐	☒	Animals and other organisms
☐	☒	Human research participants
☒	☐	Clinical data
☒	☐	Dual use research of concern

Methods

n/a	Involved in the study	
☒	☐	ChIP-seq
☒	☐	Flow cytometry
☒	☐	MRI-based neuroimaging

Animals and other organisms

Policy information about studies involving animals; ARRIVE guidelines recommended for reporting animal research

Laboratory animals	All samples used are from males. The species used in this study were chimpanzee (Pan troglodytes, 14 yo, 21 yo, 45 yo), bonobo (Pan paniscus, 36 yo, 15 yo), gorilla (Gorilla gorilla 43 yo, 51 yo), gibbon (Hylobates lar, 5 yo), macaque (Macaca mulatta, 7 yo, 9 yo), marmoset (Callithrix jacchus, 10 yo), mouse (Mus musculus, CD-1, adult; 12 h day night cycle, temperature 20-24 °C, 45-65 % humidity), opossum (Monodelphis domestica, adult), platypus (Ornithorhynchus anatinus, adult) and chicken (Gallus gallus, red junglefowl, adult) (see Supplementary Table 1).
Wild animals	Adult male platypus (Ornithorhynchus anatinus), Animals were euthanized with an intraperitoneal injection of 0.1 mg/g pentobarbital, for tissue collection. Note that the samples were collected as part of a previous study; i.e., animals were not sacrificed for the purpose of this study.
Field-collected samples	Adult male platypus (Ornithorhynchus anatinus), Animals were euthanized with an intraperitoneal injection of 0.1 mg/g pentobarbital, for tissue collection. Note that the samples were collected as part of a previous study; i.e., animals were not sacrificed for the purpose of this study.

Ethics oversight	The use of mammalian animal samples for the type of work in this study was approved by ERC Ethics Screening panels (ERC Starting Grant 242597, SexGenTransEvolution, and ERC Consolidator Grant 615253, OntoTransEvol).

Note that full information on the approval of the study protocol must also be provided in the manuscript.

Human research participants

Policy information about studies involving human research participants

Population characteristics	Human samples were obtained from official scientific tissue banks or dedicated companies; informed consent was obtained by these sources from donors prior to death or from next-of-kin. All samples are from adult caucasian males
Recruitment	Human samples were obtained from official scientific tissue banks or dedicated companies; informed consent was obtained by these sources from donors prior to death or from next-of-kin.
Ethics oversight	The use of all human samples for the type of work described in this study was approved by an Ethics Screening panel from the European Research Council (ERC) (associated with H.K.'s ERC Consolidator Grant 615253, OntoTransEvol) and local ethics committees; that is, from the Cantonal Ethics Commission Lausanne (authorization 504/12), Ethics Commission from the Medical Faculty of Heidelberg University (authorization S-220/2017), and the regional medical research Ethics committee of the capital region of Copenhagen (H-16019637)

Note that full information on the approval of the study protocol must also be provided in the manuscript.
